# Using interprofessional collaboration to reduce reported rates of central-line–associated bloodstream infection in an intensive care setting

**DOI:** 10.1017/ice.2023.279

**Published:** 2024-05

**Authors:** Hannah Musgrove, Abigail Ruby, Eman Chami, Edward Pollak, Geehan Suleyman, Arielle Gupta

**Affiliations:** 1 Surgical Intensive Care Unit, Henry Ford Hospital, Detroit, Michigan; 2 Department of Quality and Safety, Henry Ford Hospital, Detroit, Michigan; 3 Department of Anesthesiology, Henry Ford Hospital and Henry Ford Medical Group, Detroit, Michigan; 4 Department of Infectious Diseases, Henry Ford Hospital, Detroit, Michigan; 5 Department of Surgery, Henry Ford Hospital, Detroit, Michigan

## Abstract

Using a multicomponent approach that included blood-culture stewardship, evaluation for secondary sources of bloodstream infection, improved documentation, and prompt central-line removal, an interprofessional team improved patient care and reduced central-line–associated bloodstream infection rates in collaboration with the primary team on the surgical intensive care unit.

Central-line–associated bloodstream infections (CLABSIs) are preventable healthcare-associated infections (HAIs) associated with increased morbidity, mortality, and prolonged hospital stay.^1,2^ Additionally, CLABSIs are the most expensive HAIs, with an estimated cost of $46,000 per case.^3^

Surveillance criteria used to define CLABSIs in an acute-care setting are based on the National Healthcare Safety Network (NHSN) definitions.^4^ Surveillance consists of review of positive blood cultures from patients with qualifying central lines. To meet criteria for CLABSI, qualifying central lines must be in place for >2 consecutive calendar days after being accessed.^5^ A 7-day infection window period (IWP), which includes the 3 days before and after the first positive diagnostic test, is used to review infection criteria to meet the surveillance definition. For example, if a patient had a positive blood culture on January 4, the IWP would be January 1 through January 7. If no secondary sources are identified as a cause of the bloodstream infection (BSI), it will qualify as a CLABSI.^5^ Surveillance definitions are standardized to avoid variations in reporting. However, several factors, including misidentification of NHSN criteria and lack of diagnostic workup to identify secondary sources or documentation, could affect CLABSI reporting.

To understand the occurrence of CLABSIs in the surgical intensive care unit (SICU), root-cause analysis data were analyzed. Multiple opportunities that affect unit CLABSI rates were identified, including gaps in clinical assessment, diagnostic testing, blood-culture and central-line utilization, and documentation. Using an interprofessional proactive approach, we sought to reduce the number of CLABSIs by following the institutional blood-culture stewardship guideline, reducing unnecessary blood-culture and central-line utilization and evaluating for secondary sources of BSI.

## Methods

This before-and-after quasi-experimental retrospective study was completed in the SICU of an 877-bed, tertiary-care academic hospital in southeastern Michigan. The CLABSI rate per 1,000 central-line days, blood-culture order rate per 1,000 central-line days, central-line utilization ratio and BSI rate per 1,000 patient days, and standardized infection ratio (SIR) in the preintervention period (January 2019 to January 2021) were compared to the intervention period (February 2021 to December 2022). Hospital institutional review board approval and waiver of informed consent were obtained.

An interprofessional team, which included the unit medical director, infection prevention (IP) specialist, and clinical nurse specialist, reviewed electronic medical records (EMRs) of all patients with central lines as well as blood cultures ordered, They communicated findings utilizing a secure texting group. For guidance on complex cases, the team partnered with the IP medical director.

Blood-culture stewardship guidelines, based on those described by Fabre et al,^6^ were implemented in July 2019, which provided guidance on appropriate blood-culture utilization. When blood cultures were not indicated, the care team was contacted for discontinuation. When indicated, the EMR was reviewed for infection source from existing diagnostic testing and documentation. The institution implemented daily review of central-line indications in 2021, and the interprofessional team reinforced the assessment of central lines and facilitated prompt removal of unnecessary lines. Identified gaps were immediately communicated with patient care teams. Follow-up included collaborative efforts regarding proper documentation of clinical findings and treatment plan.

### Statistical analysis

The *t* test was used to compare the continuous variables and was determined statistically significant if *P* < .05. All analyses were performed using SPSS Statistics version 29 software (IBM, Armonk, NY).

## Results

The interprofessional team identified secondary BSI in 37 patients (17 in 2021 and 20 in 2022) with central lines and positive blood cultures. The CLABSI rate significantly decreased from 1.78 in the preintervention period to 0.33 in the postintervention period (*P* < .001), resulting in an 82% reduction. Similarly, the SIR declined from 1.469 to 0.279 (*P* = .001) with a resultant 81% reduction. In total, 46 blood cultures (3.3%) were discontinued in the postintervention period compared to 3 (0.1%) in the preintervention period. The blood-culture order rate was reduced by 33% (*P* < .001), and the central-line utilization ratio was reduced by 17% (*P* < .001) after the intervention was implemented. In addition, the overall unit BSI rate was 26% lower in the postintervention period (Table 1).

**Table 1.** CLABSI Rate and Standardized Infection Ratio during the Pre- and Postintervention Periods

Metrics	Preintervention Period	Postintervention Period	Reduction, %	*P* Value
CLABSIs rate per 1,000 central-line days	1.78	0.33	82	<.001
Standardized infection ratio	1.469	0.279	81	.001
Blood culture order rate per 1,000 central-line days	311	207	33	<.001
BSI rate per 1,000 patient days	13.75	10.13	26	<.001
Central-line utilization ratio per 1,000 patient days	570	471	17	<.001

Note. BSI, bloodstream infection; CLABSI, central-line–associated bloodstream infection.

This proactive approach ensured that supporting evidence was present to meet NHSN definitions for secondary BSI to avoid CLABSIs. Real-time follow-up from the interprofessional team led to early source recognition and at times redirected patient management leading to improved patient care and outcomes. The following case studies provide examples of a missed opportunity and the impact of the interprofessional team.

### Preimplementation case study

A patient presented with necrotizing soft-tissue infection. On hospital day 25, she became febrile, with blood culture positive for *Candida parapsilosis*. On day 26, she required necrotic tissue debridement. Unfortunately, tissue cultures were not obtained. On day 31, she returned to the operating room, and tissue cultures that were outside the IWP grew *C. parapsilosis*. The absence of tissue cultures during the debridement on day 26 was a missed opportunity to identify a secondary BSI during the IWP.

### Postimplementation case study

A patient presented with high fistula output. On hospital day 2, a central line was placed and imaging was completed for verification. Blood cultures were collected on day 5 when she became febrile and hemodynamically unstable. On day 6, a blood culture grew *Staphylococcus epidermidis.* The interprofessional team reviewed the EMR. A computed tomography scan from the previous hospitalization identified inflammatory changes of the spine. Findings and the IWP were communicated to the care team, who performed a targeted assessment and found the patient to be experiencing back warmth and pain. The IP medical director recommended magnetic resonance imaging. On day 7, magnetic resonance imaging findings were consistent with osteomyelitis of the spine. Thus, secondary BSI was identified, and the course of antibiotics was extended to appropriately treat the infection.

## Discussion

This project demonstrates that an interprofessional team can help decrease CLABSI rates using a multicomponent intervention that included blood-culture stewardship to (1) minimize unnecessary blood-culture orders, (2) evaluate for alternative sources of BSI, (3) improve documentation, and (4) achieve early central-line removal in collaboration with primary teams.

Dedicated unit professionals allocated to quality improvement were vital for success. Each professional role brought a distinct perspective. The team overcame several challenges and barriers. Understanding the problem, established through a review of root-cause analysis data, was the first step to success.^7^ General lack of knowledge of NHSN rules and definitions, paired with frequent changes in care teams, presented challenges. In addition, shifting the culture from reactive to proactive was a significant barrier to overcome. Consistent messaging and education were critical to improve understanding of NHSN rules, CLABSI definitions, diagnostic stewardship guidelines and central-line indications.^1^ The project was implemented during the coronavirus disease 2019 (COVID-19) pandemic, which resulted in deterioration of multiple patient safety measures and affected the incidence of HAIs, particularly CLABSIs, in various healthcare settings.^8^ Despite these challenges, the interprofessional team significantly reduced the SICUs incidence of CLABSI.

In response to this project, a CLABSI review committee was formed to identify missed opportunities during the IWP. This committee is attended by administration, unit leadership, and IP staff. Since implementation, reduction in CLABSIs has been achieved, demonstrating that this project can be implemented in other practice areas. Given the retrospective nature of this study, these results are subject to inherent limitations. Additionally, clinical outcomes during the intervention were not measured.

Significant reductions in CLABSI and BSI rates, SIR, blood-culture orders and central-line utilization were observed when using interprofessional collaboration. Institutions should consider implementing this intervention to reduce CLABSI rates.
